# The Importance of Proper Oxygenation in 3D Culture

**DOI:** 10.3389/fbioe.2021.634403

**Published:** 2021-03-30

**Authors:** Hubert M. Tse, Graeme Gardner, Juan Dominguez-Bendala, Christopher A. Fraker

**Affiliations:** ^1^Department of Microbiology, The University of Alabama at Birmingham, Birmingham, AL, United States; ^2^Department of Surgery, Diabetes Research Institute, Leonard M. Miller School of Medicine, University of Miami, Coral Gables, FL, United States; ^3^Department of Cell Biology and Anatomy, University of Miami Miller School of Medicine, Miami, FL, United States

**Keywords:** 3D culture, spheroids, oxygenation, gas-permeable, hypoxia, hyperoxia (oxygen)

## Abstract

Cell culture typically employs inexpensive, disposable plasticware, and standard humidified CO_2_/room air incubators (5% CO_2_, ∼20% oxygen). These methods have historically proven adequate for the maintenance of viability, function, and proliferation of many cell types, but with broad variation in culture practices. With technological advances it is becoming increasingly clear that cell culture is not a “one size fits all” procedure. Recently, there is a shift toward comprehension of the individual physiological niches of cultured cells. As scale-up production of single cell and 3D aggregates for therapeutic applications has expanded, researchers have focused on understanding the role of many environmental metabolites/forces on cell function and viability. Oxygen, due to its role in cell processes and the requirement for adequate supply to maintain critical energy generation, is one such metabolite gaining increased focus. With the advent of improved sensing technologies and computational predictive modeling, it is becoming evident that parameters such as cell seeding density, culture media height, cellular oxygen consumption rate, and aggregate dimensions should be considered for experimental reproducibility. In this review, we will examine the role of oxygen in 3D cell culture with particular emphasis on primary islets of Langerhans and stem cell-derived insulin-producing SC-β cells, both known for their high metabolic demands. We will implement finite element modeling (FEM) to simulate historical and current culture methods in referenced manuscripts and innovations focusing on oxygen distribution. Our group and others have shown that oxygen plays a key role in proliferation, differentiation, and function of these 3D aggregates. Their culture in plastic consistently results in core regions of hypoxia/anoxia exacerbated by increased media height, aggregate dimensions, and oxygen consumption rates. Static gas permeable systems ameliorate this problem. The use of rotational culture and other dynamic culture systems also have advantages in terms of oxygen supply but come with the caveat that these endocrine aggregates are also exquisitely sensitive to mechanical perturbation. As recent work demonstrates, there is a strong rationale for the use of alternate *in vitro* systems to maintain physio-normal environments for cell growth and function for better phenotypic approximation of *in vivo* counterparts.

## Introduction

Biomedical research of 3D cell culture has been ongoing for decades, increasing dramatically with the recent boom in stem cell research and associated cellular therapies (Moscona A., 1961; Knisely et al., 1969; Bruland et al., 1985; Li et al., 1992; De Moor et al., 2018; Gargotti et al., 2018; Jokinen et al., 2020; Qadir et al., 2020). Early seminal research focused on tumor spheroids and the generation of *in vitro* models to test therapies against the malignant cells (Inch et al., 1970; Bruland et al., 1985; Vescio et al., 1987). These early works spawned expansive research into basic biological mechanisms such as proliferation, differentiation, cell death, and nutrient/metabolite requirements in culture (Moscona A.A., 1961; Knisely et al., 1969; Carlsson et al., 1979; Gille and Joenje, 1992; Matta et al., 1994; Singhvi et al., 1994; Bader et al., 1996; Gassmann et al., 1996; Groebe, 1996).

Resoundingly, the majority of early research found dramatic improvements with 3D culture compared with 2D culture of the same cells. Simultaneous molecular and functional analysis of 2D and 3D cultures led to the discovery that 3D cultures tend to more accurately recapitulate the *in vivo* environment in cell shape/structure and environment. This, in turn, influences gene/protein expression and cell function. As a result, 3D cultures traditionally behave more like their *in vivo* counterparts. Despite dramatic improvements in expression and function, a substantial gap exists between 3D aggregates/spheroids and the *in vivo* organoids they are meant to model. We posit that one potential explanation for these observed differences is the variability in employed culture methods, particularly related to tissue oxygenation. Through years of foundational research and technological advances, the limitations of tissue oxygenation in culture methods have been described and the critical importance of variables such as cell seeding density, oxygen consumption rate (OCR), growth rate, and media height have been described (Carlsson et al., 1979; Mueller-Klieser and Sutherland, 1982; Heacock and Sutherland, 1986; Mueller-Klieser et al., 1986; Freyer et al., 1991; Gassmann et al., 1996; Groebe and Mueller-Klieser, 1996; Papas et al., 2005; Grayson et al., 2006; Fehrer et al., 2007). Due to the urgency with which all living cells require oxygen for the maintenance of metabolic processes, much focus has shifted to addressing insufficiencies in cell culture either through the design of culture systems based on convective flow and improved oxygen transfer through culture surfaces or by tissue engineering approaches to improve vasculogenesis.

For years, static culture has been performed in gas impermeable systems where steep oxygen and nutrient gradients form, shifting cells/aggregates down alternate pathways of metabolism, growth, and differentiation. Recent work from many groups, including our own, demonstrates that proper oxygenation can dramatically alter the viability and function of cell cultures, particularly of 3D tissues, resulting in an even more physio-normal approximation of their *in vivo* counterparts (Carlsson et al., 1979; Tuncay et al., 1994; Saini and Wick, 2004; Wang et al., 2005; Fraker et al., 2007, 2009, 2013; Powers et al., 2008, 2010; Simon and Keith, 2008; Anada et al., 2012; Cechin et al., 2014). This article will examine the role of oxygen in the growing area of 3D culture for therapeutic cell-based applications as it pertains to both primary endocrine cell aggregates (islets of Langerhans) and endocrine stem cell aggregates (SC-β). The utility of finite element modeling (FEM) as a way of optimizing culture settings to target physiologically relevant tissue oxygen concentration/partial pressures (pO_2_ in millimeters of mercury, relative to atmospheric pressure, 760 mmHg) will be highlighted. Particularly, we will examine alternate culture methods utilized historically, prior to the advent of efficient computational modeling, and will examine by retrospective FEM the oxygen profiles in these systems to determine the role of tissue oxygenation in some of the experimental outcomes. FEM can also dramatically reduce experimental waste in terms of materials and labor hours making it an efficient method for designing and validating experiments.

Importantly, tissue oxygenation in both 2D and 3D culture in various devices has been well-studied but the majority of these studies have been focused on conventional oxygen concentrations (95% RA) or extremely hypoxic conditions <4% O_2_ and primarily in gas impermeable plastic systems (Knisely et al., 1969; Mueller-Klieser and Sutherland, 1982, 1984; Mueller-Klieser, 1984; Mueller-Klieser et al., 1986; Acker et al., 1987; Deshpande and Heinzle, 2009; Murphy et al., 2017). The conclusions of many such studies in 3D culture demonstrate the steep gradient formation and tissue volume anoxia that is expected in plastic systems. The advent of membrane-based culture systems and methods for improving oxygen delivery to the cell culture surface has created a paradigm shift where historical studies are being repeated under more physiological oxygenation with pronounced differences in cellular viability and function.

## Cellular Oxygen Demand

Mammalian development occurs at a partial pressure of oxygen (pO_2_) governed by mass transfer prior to tissue vascularization (Simon and Keith, 2008). Mass transfer limitations dictate that ∼ 0.5–1 mm^3^ is the maximal volume that can be adequately oxygenated by simple diffusive permeability, dependent on the tissue specific OCR and the local niche pO_2_ (Colen et al., 1999; McGrath et al., 2003). Therefore, much tissue development occurs at a pO_2_ approximating anoxia and this is the time period when much of the characteristic tissue proliferation is observed. With the advent of blood flow, pO_2_ increases to tissue specific ranges from 3.8 to 100 mmHg with little variation across much larger, perfused regions.

Cellular OCR in the literature ranges between <1 and 350 × 10^–18^ mol/s. This is in line with the oxygen requirements needed for the metabolism of 2,500 kcal per day which requires approximately 22 mols of oxygen or 2.5 × 10^–4^ mol/s. Assuming the commonly accepted number of cells in the adult human body of 37.2 trillion, this equates to an average 6.72 × 10^–18^ mol/cell s^–1^ with variation due to metabolic demand and cell function. Based on an average human cell diameter of 20 μm, this translates to an average rate of 1.6 × 10^–3^ mol/m^3^ of tissue s^–1^ (Wagner et al., 2011).

Historically, both 2D and 3D cell culture protocols have utilized gas-impermeable plastics kept within humidified incubators containing 95% room air (RA) and 5% carbon dioxide (CO_2_). Accounting for vapor pressure differences, the standard incubator oxygen partial pressure is approximately 142 mmHg. However, there is much variability in utilized methods related to cell seeding density and media height, further exacerbated by the range of OCR associated with mammalian cells. Particularly, different culture platform geometries necessitate variations in media height to prevent evaporation and provide adequate nutrients. These variations, exhibited in Table 1, can be detrimental to cultured cells, especially those with higher OCR.

**TABLE 1:** Inherent variations in oxygen diffusion distances (culture medium height) due to the dimensions of typically utilized plastic cell culture systems.

Culture system	Culture surface area (cm^2^)	Typical culture medium volume range (mL)	Culture medium height range (mm)
96-well	0.32	0.1–0.2	3.12–6.25
24-well	1.9	0.5–1	2.63–5.26
6-well	9.6	1–3	1.04–3.13
T-75 flask	75	8–15	1.07–2
T-175 flask	175	35–53	2–3.03

*1D oxygen diffusion from the air/liquid interface varies from 1.04 to 6.25 mm depending on dimensions and culture medium volume.*

## 2D vs. 3D Culture: Endocrine Cells and Clusters as an Example

The above variables can affect both 2D monolayer cultures and, more so, 3D aggregates resulting in prolonged exposure to elevated oxygen levels that can serve as a substrate for the formation of toxic free radical species on one extreme and pronounced hypoxia/anoxia, necrosis and impaired function on the other extreme (Kazzaz et al., 1999; Martens et al., 2005). Particularly, the high OCR and 3D geometry of endocrine aggregates (islets of Langerhans, stem cell-derived beta cells) restrict them to a low seeding density and culture medium height of ∼1.3 mm. These cells and clusters have reported OCR most often ranging from 1 to 5.0 × 10^–2^ mol/m^3^ s^–1^, more than an order of magnitude higher than the cellular average. This is not surprising given the critical role they play in the production and storage of multiple hormones and the tight regulation of glucose homeostasis (Papas et al., 2000; Murray et al., 2005; Pi et al., 2007; Buchwald, 2009, 2011; Fraker et al., 2013; Suszynski et al., 2014b; Qadir et al., 2020).

**FIGURE 1:**
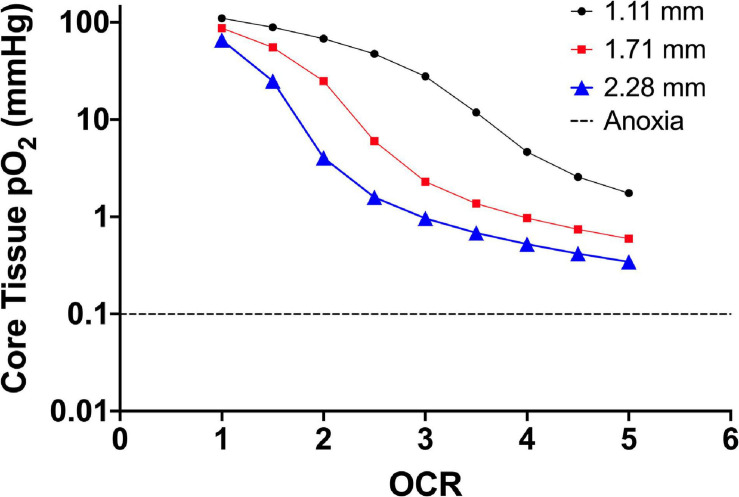
Core tissue oxygen profile of 2D monolayer (20 μm cell diameter) as a factor of culture media height (three different line plots) and increasing OCR (along the *x*-axis). The decrease in core pO_2_ corresponds to an increase in OCR along the *x*-axis and the downward shift in core pO_2_ is a result of increased media height. The OCR are representative of the published range of most primary endocrine somatic cells and cell lines. The dashed line shows the accepted theoretical cutoff for tissue anoxia and eventual necrotic death in endocrine aggregates.

Figure 1 is finite element modeling (FEM) of 2D monolayer cultures showing the pO_2_ at the core region across a parametric sweep of OCR relevant to endocrine cells and clusters examining the effect of culture media height on tissue pO_2_.

From the model, 2D monolayer culture with 100% confluence is not prone to anoxia. In fact, at oxygen consumption rates at the lower end of the presented range, the pO_2_ experienced by the cells is “supra-physiological.” This could explain reports with culture at reduced environmental pO_2_ that results in improved proliferation and viability, as the lower external pO_2_ better represents the physiological niche of the cells (Fraker et al., 2007; Powers et al., 2008, 2010; Millman et al., 2009; Cechin et al., 2014). Using an oxygen consumption rate of 3.0 × 10^–2^ mol/m^3^ s^–1^ as a reference value, simply changing the media height by ∼1 mm shifts the core tissue pO_2_ nearly 30 mmHg (27 mmHg at 1.11 mm height to 0.9 mmHg at 2.28 mm height). As diffusion distances in vascularized tissues are typically not more than 5–10 μm, there are limited gradients that form *in vivo* related to not only oxygen but also most metabolites and waste products. The extent to which the gradients in culture affects cell function and viability, on the other hand, is not fully known.

In 3D cultures, core anoxia is common due to increased diffusion distances. This is evident in islets and islet-like clusters with sizes typically ranging from 50 to 400 μm. Islets above 100 μm cultured on plasticware experience increasing anoxia and loss of secretory capacity dependent primarily on oxygen consumption rate and media height and less so, related to seeding density. With a typical oxygen consumption range of 1.0–5.0 × 10^–2^ mol/m^3^ s^–1^, islets are limited to a seeding density that is well-established in the literature of about 3% of the culture surface area and a media depth not more than 1.1–1.3 mm (Papas et al., 2005; Avgoustiniatos et al., 2008; Kitzmann et al., 2014). As islet/endocrine spheroid viability and function are exquisitely dependent on tissue pO_2_, deviations from the culture recommendations can result in pronounced islet/endocrine cluster loss during culture. In fact, it has been demonstrated in the literature that apoptosis in cultured islets directly correlates with expression of HIF-1a (Moritz et al., 2002). This results in increased costs related to islet culture as a typical human islet preparation of approximately 250,000 islet equivalents (islet size scale correction to mean diameter of 150 μm; IEQ) requires 10–12 T175 flasks. Early work by the group of and Colton, Papas, Avgoustiniatos, and Dionne utilized computational modeling to investigate the oxygenation limitations in conventional culture methods for islets of Langerhans (Dionne et al., 1989, 1991, 1993, 1996; Papas et al., 2005). Through their detailed study of OCR in endocrine cells and spheroids, they identified the K_*m*_ value associated with islet OCR (0.4 mmHg), and pO_2_ values associated with anoxia (∼0.1 mmHg and below) and impaired insulin secretion (∼2.5 mmHg and below). They continued this important work with further in-depth study of (a) the use of OCR as a metric of islet potency, (b) limitations in encapsulation devices and *in vivo* cellular replacement therapies in Type 1 Diabetes Mellitus (T1D), and (c) the development of gas permeable culture systems to address oxygen limitations (Avgoustiniatos et al., 2008; Kitzmann et al., 2014; Suszynski et al., 2014a, 2016; Papas et al., 2016; Cao et al., 2020). In one seminal paper by the group, FEM was performed assuming the standard IEQ diameter of 150 μm cultured in a square array and varying the culture density and culture medium volume to maintain the 1,000 IEQ/mL culture standard. Three different oxygen consumption rates were evaluated. In addition to plastic, culture was also simulated on 275 μm silicone membranes. They observed precipitous increases in anoxia on plastic culture platforms relative to OCR and the combined effect of culture density/medium depth (Papas et al., 2005).

The equivalent scaling originated from hand-counts of numerous islet preparations (rodent, dog, non-human primate, porcine, and human) demonstrating that the size bin with the largest tissue volume percentage was ∼150 μm. Our group confirmed this in studies comparing automated counters with hand counting protocols (Buchwald et al., 2009). Table 2 shows representative tissue volume distributions across size bins from 184 human islet preparation hand-counts.

**TABLE 2:** Tissue volume percentages from 184 human islet preparations broken into IEQ counting size bins.

Size bin	50–100	100–150	150–200	200–250	250–300	300–350	350–400	>400
Mean	7.34%	15.23%	21.1%	16.27%	14.47%	12.40%	9.1%	4.07%
SEM	0.50%	0.70%	0.70%	0.70%	0.90%	0.90%	0.90%	0.70%

*The bins centered around 150 μm account for nearly 37% of the total preparation volume, the rationale behind the 150 μm IEQ size.*

The rationale behind the IEQ standardization is clear from the ∼37% of the tissue volume represented by the bins centered around 150 μm. However, over 60% of the tissue volume is above 150 μm which leads to underestimation in anoxic volume in the tissue preparations. To better represent this, our group generated FEM models to represent anoxia in the full islet preparation volume. After solving, anoxic volume percentages were calculated for each size range and then total anoxic tissue volume percentage was determined by the following equation:

$$\%A_{total}=\sum_{50-100}^{>400}\%A_{size}\times \%V_{size}$$

where, %A_*total*_ is total volume percentage of anoxic tissue, %A_*size*_ is the percentage of anoxic tissue volume determined by FEM for each islet size bin (Table 2) and %V_*size*_ is the mean volume percentage of tissue for each islet size bin (Table 2).

**FIGURE 2:**
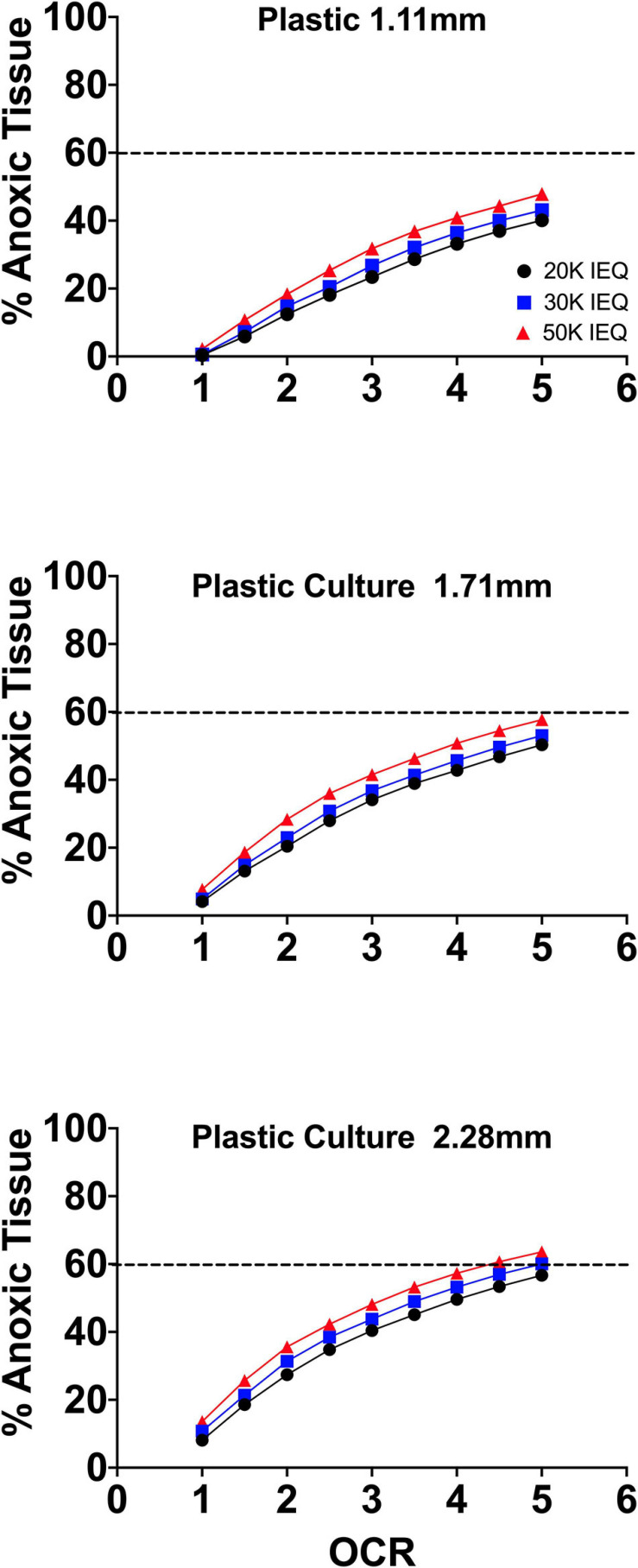
FEM results of islet spheroids in standard culture (95% RA/5% CO_2_; plastic surface; 37°C) with varied seeding density and culture medium height across the range of reported islet oxygen consumption rate. Tissue anoxia is observed, primarily in IEQ sizes >100 μm, even at low oxygen consumption rates and is significantly affected by culture medium height (∼Δ20% min to max) and increased OCR (∼Δ50% min to max) and less so by seeding density (∼Δ7% min to max).

Figure 2 summarizes the effects of the oxygen consumption rate range, three conventional medium height variations (20, 30, 50 mL in a T-175 flask; 1.11, 1.71, and 2.28 mm, respectively) and three seeding densities (20, 30, and 50,000 IEQ in a T-175 flask; 2.76%, 3.67%, and 5.05%, respectively) on islet/islet-like cluster anoxia on standard plastic culture devices.

The models confirm the observed and previously modeled deficiencies of islet/endocrine spheroid culture on plastic systems. Tissue anoxia is observed in IEQ > 100 μm heavily dependent on OCR and culture medium height and less so on seeding density, albeit still a factor.

Despite well-documented culture suggestions and supportive FEM studies from multiple groups, there are still broad deviations in the literature in both islet/endocrine spheroid seeding density and culture medium height that could explain reported variations in pre/post-transplant function and viability. There is a clear need for culture standardization and cost-effective methods for improving culture oxygenation. Recent work has focused on addressing these challenges for the scale-up and implementation of clinical cell-based therapies.

## Methods to Improve Oxygenation in 3D Culture: Endocrine Clusters as an Example

Early islet research groups including those of Colton, Kevin Lafferty and islet isolation pioneer, Paul Lacy, understood the importance of oxygen limitations in culture from early modeling and observed loss of function and viability (Bowen et al., 1980; Dionne et al., 1989, 1991). They explored various methods to improve oxygenation ranging from increasing incubator oxygen concentration to levels as high as 95%, low temperature culture, perfusion systems, and gyroscopic/rotational culture (Scharp et al., 1978; Ono et al., 1979; Lacy et al., 1982). All of these methods ameliorated tissue hypoxia/anoxia but had associated challenges and beneficial unexpected findings.

### Increasing External Oxygen Concentration

In studies first by the group of Lafferty and then by Lacy et al. (1982) culture in elevated incubator pO_2_ resulted in a stark depletion of tissue-resident immune cells present in islets. These cells surveil and relay information about islet well-being to immune response elements. Additionally, the extended culture resulted in aggregation of individual IEQ into “mega-islets.” The depletion of the tissue-resident immune cells in elevated oxygen resulted in significantly prolonged islet engraftment in allogeneic and concordant xenogeneic transplant settings (Bowen et al., 1980).

In one paper by Bowen et al. (1980), they cultured 50 IEQ in 35 mm dishes with 2 mL of culture medium, initially, moving to 0.75 mL after the first of three culture medium changes in the 4–7 days culture period. Incubator gas concentration was 95% O_2_ and 5% CO_2_. After the first change of culture medium, they also began to observe aggregation of the IEQ into one large “mega-islet.” Interestingly, IEQ that failed to aggregate did not survive the culture at elevated oxygen concentrations suggesting that the aggregation may result in a “mega-islet” pO_2_ that better approximates standard culture or *in vivo* levels.

In another paper by Lacy et al. (1982), they modified the 2-stage protocol of Lafferty by first culturing 50 IEQ/well in U-bottom 96-well plates with 200 μL of culture medium. This significantly increased the culture medium height from ∼2 mm in the Lafferty protocol to ∼6.2 mm for the first stage of the culture progressing to culture on a 35 mm dish with 0.75 mL of culture medium, as in the Lafferty protocol.

**FIGURE 3:**
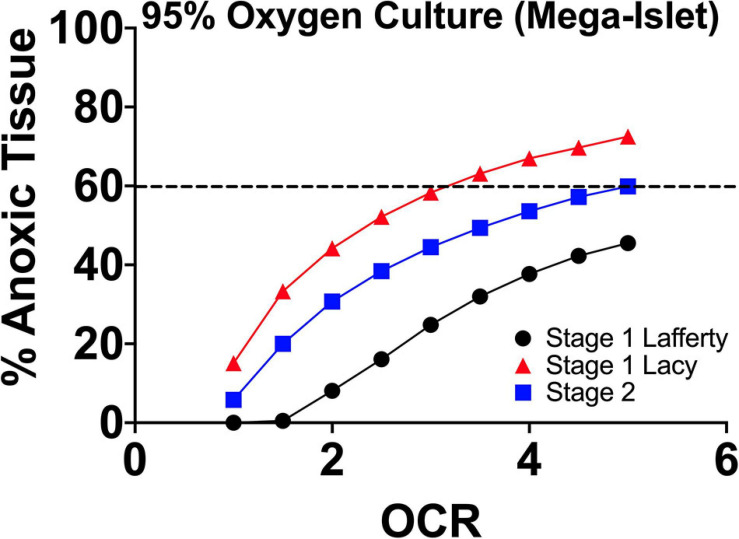
FEM anoxia results of two stage mega-islet cultures (95% O_2_/5% CO_2_; plastic surface; 37°C) from the group of Lafferty and Lacy, respectively. Prior to aggregation (Stage 1), the tissue anoxia is lower at all oxygen consumption rates but with aggregation, anoxia become significantly higher throughout the tissue given the increase in tissue geometry. The elevated pO_2_ maintains non-anoxic tissue at levels approximating culture of IEQs of standard size range (50–400 μm) and seeding density. At the same time, the supra-physiological pO_2_ depletes the tissue resident immune cells.

In Figure 3, using the culture parameters described in the papers of Lafferty and Lacy, we performed FEM of the estimated anoxia during 2-stage mega-islet culture in order to better understand the effects of culture oxygenation on the experimental observations.

The increased pO_2_ sustains the larger tissue dimensions at a level of hypoxia/anoxia approximating the culture of IEQ of standard size range and seeding density. There is no apparent oxygenation benefit but the elevated pO_2_ allows for IEQ aggregation and reduction of tissue-resident immune cells. The thought of both groups was that increased oxygen was responsible for the loss of tissue-resident cells, but the presence, indicated by FEM, of similar anoxic tissue volumes to standard 95% RA/5% CO_2_ cultures suggests that hypoxia/anoxia may also play a role. In both research efforts, when these mega-islets were utilized in sub-renal capsule transplants in chemically induced diabetic recipients, they improved allogeneic/concordant xenogeneic graft longevity without concurrent immunosuppression. This suggests that potentially (1) the tissue-resident cells play a critical role in graft rejection signaling recipient immune responses and (2) extended culture reduces antigen shedding and thereby, proinflammatory immune responses.

**FIGURE 4:**
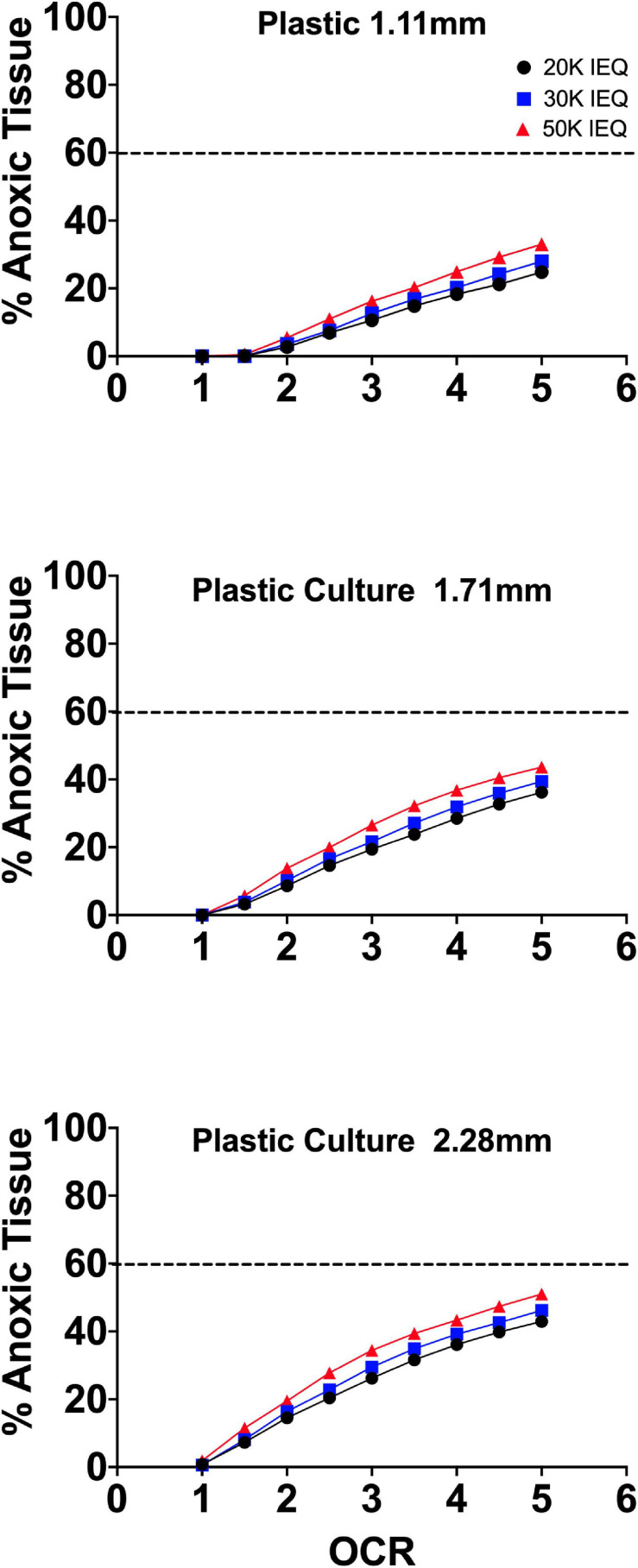
FEM of standard islet culture on plastic with an external oxygen concentration of 35%. The modeled anoxia was ∼50–70% relative to culture at 20% O_2_.

Our group also implemented increased oxygen levels in standard islet culture examining islet function and viability. FEM of this approach is shown in Figure 4. The modeled reduction in tissue anoxia was 30–50% compared to conventional 20% O_2_. This did not translate to significant *in vitro* functional improvements. This could be the result of supra-physiological concentrations in smaller islets (100, 150 μm) resulting in increased oxidant concentration and potentially, free-radical mediated damage to secretory capacity. These high oxygen levels were not experienced by seeded IEQ in the prior mega-islet experiments due either to increased media depth or closer IEQ seeding proximity. Clearly, there is a balance of proper oxygenation where both too much and too little are detrimental, and the multiple variables involved further complicate efforts to adequately maintain endocrine clusters.

### Culture on Liquid Permeable Transwell Devices

Another approach that has been increasingly implemented in the culture of 3D aggregates, including islets and endocrine precursors/pancreatic progenitors is the use of transwell devices. These come ready to use in most plate well geometries. 3D aggregates are suspended on a liquid permeable membrane raised off of the basal plastic surface of the culture well ∼1 mm. Simply elevating the culture above the gas impermeable surface and allowing for mass transfer on both the basal and apical surface of the tissue improves the oxygen profiles. The apical surface is covered by a minimal height of culture medium reducing oxygen mass transfer distances but requiring frequent changes to prevent tissue drying due to evaporative loss (Bigas et al., 1995; Uroic et al., 2010).

**FIGURE 5:**
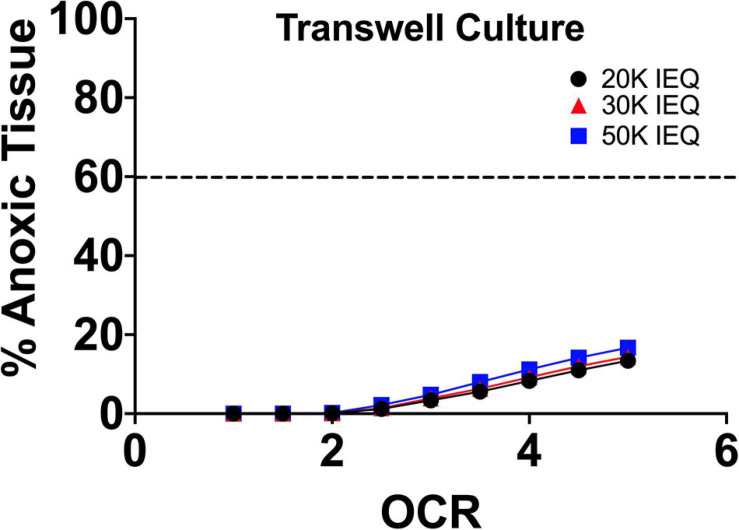
FEM of islet culture on transwell surfaces. The reduction of culture medium height due to closer proximity of the islets to the liquid/gas interface and the imposed distance from the gas impermeable basal surface (plastic) results in significant drops in tissue hypoxia/anoxia at all oxygen consumption rates.

As can be seen in FEM (Figure 5), the elevation of the tissue above the gas impermeable plastic and closer proximity to the liquid/gas interface causes a significant reduction in tissue hypoxia. Limited device geometries, however, evaporative losses/tissue dehydration at the apical surface and improved culture alternatives have made this approach recently less utilized.

### Low Temperature Culture

**FIGURE 6:**
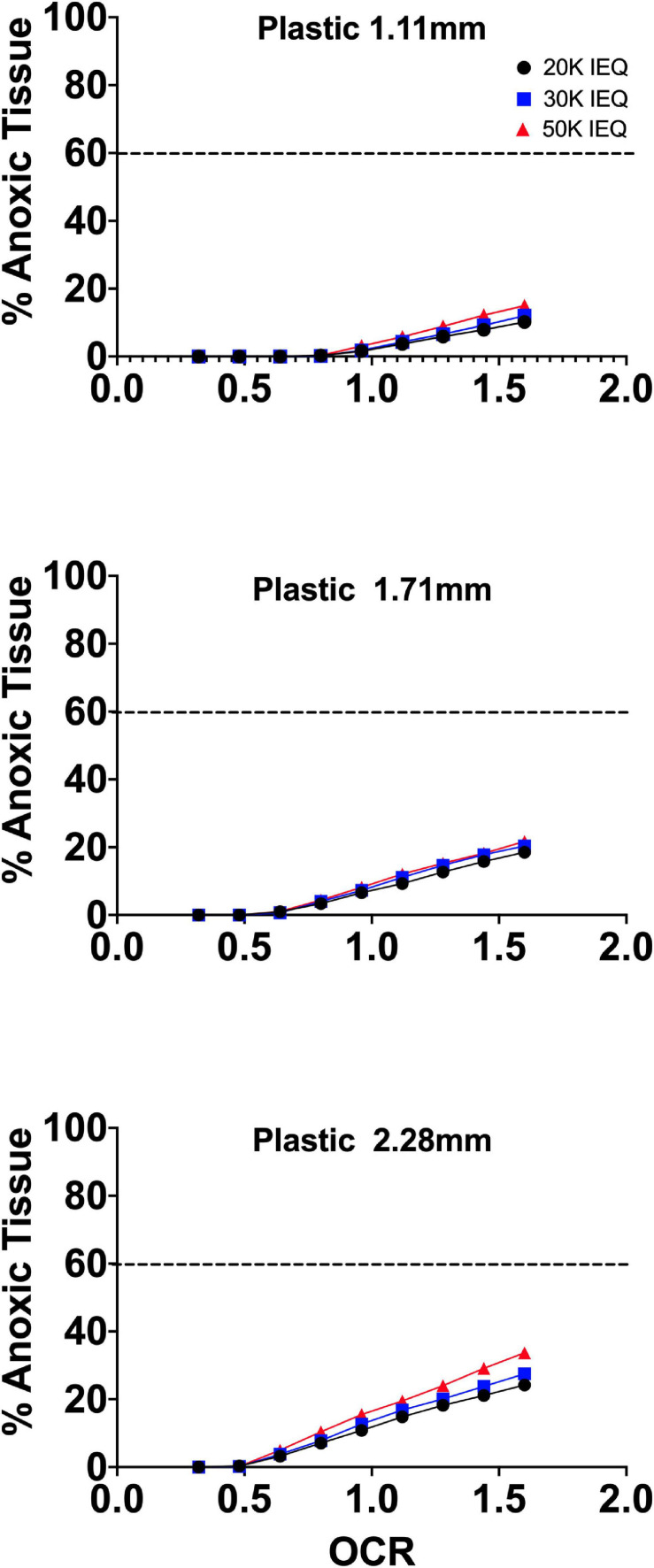
FEM of low temperature islet culture on conventional plastic culture systems. The reduction of temperature significantly reduces tissue anoxia due to the relative Arrhenius drop in oxygen consumption rate (68%) indicated along the *x*-axis (0.32 × standard islet OCR values, 1.0–5.0 × 10^−2^ mol/m^3^ s^−1^). Tissue anoxia was 20–35% less in low temperature culture relative to standard culture settings and dependent on seeding density and culture medium height.

Another early and continued approach to prevent post-isolation IEQ loss and anoxia is low temperature culture (20–25°C). Started in the late 1970s by the research groups of Lacy, Talmage and others, low temperature culture was observed to reduce antigenicity and immune response, improve function and viability and prevent anoxia and culture losses (Chase et al., 1979; Ono et al., 1979). The benefit of the low temperature culture comes from the reduction in oxygen consumption and metabolic activity of the endocrine clusters, which follow an Arrhenius behavior. These reaction rates display an exponential relationship to temperature described by the equation:

$$Rate=Rate_{0}e^{\frac{-E_{A}}{RT}}$$

where, Rate_0_ is a pre-exponential reaction factor, E_*A*_ is the activation energy for the metabolic reaction, R, the universal gas constant (8.314 mol⋅kJ^–1^ K^–1^) and T, the temperature in degrees Kelvin. For reactions such as oxygen diffusion in the system, this also results in an exponential decrease in diffusivity. This drop is countered by an increase in dissolved oxygen content (solubility) resulting in a minimal change in oxygen transfer, overall. The exponential drop in oxygen consumption rate (60–70%), however, has a pronounced effect on the tissue oxygen distribution and this significantly reduces the volume of tissue affected by hypoxia/anoxia. This can be seen in the FEM analysis in Figure 6.

Overall, tissue anoxia was reduced by 20–35% in low temperature culture relative to standard culture settings and dependent on seeding density and culture medium height. It is not surprising that this reduction in tissue loss would translate to improved function and viability upon return to physiological temperature and would result in reduced antigen shedding and recipient immune response, as was observed when transplanted.

### Bioreactors, Gyrotational Culture and Convective-Flow Culture Systems

It is clear from the FEM of various static culture settings that oxygen consumption rate, plating density and tissue diameter are critical variables that are often disregarded in culture design. Additionally, pO_2_ gradients range from surface to core values that are non-physiological and this can adversely affect tissue function and viability. This is more prevalent in 3D cell aggregates and presents a substantial obstacle to scale-up and implementation for therapeutic cell replacement applications. Given that most historical research has been performed using plastic culture systems and using immortalized cell lines, oxygen limitations have been frequently overlooked. Recently, as culture is moving toward physiologically relevant 3D aggregates, the limitations of oxygenation in plasticware have become apparent and there is a growing body of engineering work in the development of alternative culture systems for enhanced oxygenation.

Initially used in scale-up production of fermentation processes and the production/synthesis of medicinal substances from biological reactions (e.g., insulin production from recombinant DNA), bioreactors have been further engineered for a multitude of applications. Newer systems have varied device geometries and real-time controllers for atmospheric gas concentrations, fluidics, and temperature. The advantage of these systems is that they have the potential to eliminate pO_2_ gradients within the culture medium either by continued supply of fresh medium or through constant mixing in contact with the liquid/air interface.

In the context of islets and endocrine aggregates/SC-β, flow systems have long been in use. Early work in the laboratory of Paul Lacy utilized gyrotational culture devices to preserve isolated islets in collaborations with NASA and McDonnell Douglas examining the effects of rotation and microgravity on islet isolation, function, and viability (Buitrago et al., 1977; Scharp et al., 1978; Britt et al., 1981). These devices employed rotation along multiple axes to prevent islet settling onto basal, gas-impermeable culture surfaces and to continuously bathe the islets in well-mixed culture medium. These and further studies in microgravity culture systems with isolated islets demonstrated reduced immunogenicity and improved viability and function over long-term culture periods (Rutzky et al., 2001, 2002; Tobin et al., 2001; Luca et al., 2006).

Spinner flasks aid in the formation of 3D aggregates from single cells by maintaining rotational flow driven by magnetic stirrers. Many systems come with ports for easy gas exchange and media sampling/replacement. Dependent on cell density, rotational speed and time of culture, uniform clusters of a desired spherical geometry can be readily formed. These systems have been utilized in the generation of 3D islet-like spheroids from immortalized beta-cell insulinoma lines (MIN-6) exhibiting improved viability, proliferation and insulin secretion (Lock et al., 2011).

As SC-β production has scaled for pre-clinical and clinical work, high-throughput and large volume culture has dictated the transition from inefficient static culture systems to flow systems, primarily spinner flasks/bioreactors. The groups of Doug Melton and Semma Therapeutics along with former lab members, such as Jeffrey Millman, have established differentiation culture protocols implementing rotational devices to successfully generate reproducible large-scale batches of SC-β (Hrvatin et al., 2014; Pagliuca et al., 2014; Millman et al., 2016; Vegas et al., 2016; Peterson et al., 2020; Helman and Melton, 2021). Of note, until the recent paper by the group of Yoshihara et al. (2020) these aggregates relied on steps of *in vivo* terminal differentiation and a delayed function greater than the 5 days reported in the recent paper (Yoshihara et al., 2020). This delayed response has been attributed by Davis and Melton to a lack of anaplerotic cycling resulting in inefficient glucose responsiveness. This was reversed by exposing the SC-β to metabolites from late glycolysis and intermediate stages of the citric acid cycle (Davis et al., 2020). The SC-β generated by Yoshihara et al. (2020) displayed increased oxidative metabolism with oxygen consumption rates similar to healthy primary islets indicating, perhaps, that the early *in vivo* function was due to proper anaplerotic cycling not observed in the work of Davis and Melton. It should be noted that the entire differentiation procedure of the Evans group was performed on gas-impermeable plastic systems with a basal methylcellulose layer while the Davis and Melton paper utilized planar followed by suspension rotational culture, per their standard protocols.

Culture bags on mechanical rockers can also be used for 3D aggregates, but typically of pre-formed aggregates. In this case, the cells are bathed in culture media that is gently agitated in a rocking motion across the cells (Singh, 1999; Tsai et al., 2017). Their utilization with islets and endocrine spheroids has been limited, however (Schmied et al., 2000). As with spinner flasks, the more advanced systems come with controllers for gas concentration, temperature, fluidics, and contain ports for easy addition and sampling of culture media. Additionally, more advanced spinner flask and rocker systems come with a variety of sensors measuring not only dissolved gas concentration, but also glucose consumption, lactate production and pH of the culture medium.

As would be expected, these systems are ideal for oxygenation throughout the culture environment external to cell clusters/tissue maintaining a uniform tissue surface pO_2_. This system completely prevents anoxia in the relevant size range of islets (50–400 μm) across the range of typical oxygen consumption rates (1.0–5.0 mol/m^3^ s^–1^). In fact, even at tissue geometries approaching 750–1000 μm, anoxia is less than experienced by islets of standard size range (50–450 μm) in standard plastic culture.

While ideal for preventing hypoxia/anoxia, bioreactor systems are often cumbersome, require additional equipment, such as magnetic stir plates and interfaces for sensors and gas lines and can increase reagent utilization. Importantly, bioreactors and their associated convective-flow devices can increase the chance for contamination due to needed manipulation and additional component interfaces. The systems have also been shown to generate undesirable shear forces and bubbles that can have adverse effects on cells/tissues. The critical scalar factor related to adverse effects of shear is energy dissipation rate (EDR) expressed in units of W/m^3^. In a paper by Shenkman et al. (2009) shear forces on islets of Langerhans in flow culture systems appeared to have minimal effect on viability and function as measured by Caspase activity and oxygen consumption rate (Shenkman et al., 2009). This was observed up to a volumetric flow rate of 50 mL/min translating to an EDR of 9,600 W/m^3^. As reference, a spinner flask at a maximal rotation of 200 rpm has an EDR of about 1,500 W/m^3^ while bubble rupture or passage through a 200 μL pipette tip in 0.2 s have an EDR of approximately 1 × 10^5^ W/m^3^. This paper did not examine secretory function, however, which could be more informative in understanding the subtleties of shear effects as most papers in the field examine endpoints like flow induced necrosis or lysis and not functional effects.

More recent publications utilizing microfluidic systems have demonstrated that shear forces with EDR well below the levels of spinner flasks impair calcium channel mediated insulin secretion in surface cells of islets (Sankar et al., 2011; Silva et al., 2013). The lack of definitive understanding of the effects of fluidic shear on islet/endocrine spheroid function and viability could explain some of the unexpected outcomes in large-scale SC-β culture protocols, such as delays in terminal differentiation and function. Given the near-clinical stage of this cellular replacement research, there is a need for further detailed study into the range of effects of shear forces on endocrine spheroid viability and function.

### Microfluidic Culture Platforms

A recent trend in islet/SC-β culture is the use of microfluidic or lab-on-a-chip culture systems. These platforms assimilate multiple functions into a single unit utilizing flow-based culture. They typically have geometries on the order of square millimeters to centimeters and due to reduced reagent consumption, they can dramatically shrink experimental costs. They are also typically designed with an array of sensors for longitudinal monitoring of temperature, fluid flow/pressure, gas concentration and other pertinent parameters to a specific cell type. They can be used to monitor cellular feedback, for imaging analysis and for biochemical assays, like glucose-stimulated insulin release with islets. The fluidic inputs allow for the introduction of an unlimited array of compounds making the platform useful for pharmaceutical studies and the development of differentiation/culture protocols. Importantly, these platforms allow for the study of compartmentalized cells and tissues to better understand the physiological interactions of, for example, vascular or immune cells with other specific somatic tissues, like islets.

In the context of islet/SC-β research, lab-on-a-chip platforms have been used in select studies examining the prevention of endothelial cell loss in islets post-isolation, dynamic imaging studies, insulin/glucagon/somatostatin secretion studies, differentiation of human embryonic stem cells into SC-β and endocrine spheroid microencapsulation (Sankar et al., 2011; Silva et al., 2013; McMillan et al., 2016; Nourmohammadzadeh et al., 2016; Lenguito et al., 2017; Sharma et al., 2017; Lee G. et al., 2018; Jun et al., 2019). These novel systems are remarkable for small-scale discovery research but one stark omission in most studies has been a comparison to conventional culture methods to see if these systems improve endocrine spheroid viability or function. Additionally, scale-up of these fluidic systems to manage whole islet preparations or batches of SC-β would likely be costly and difficult relative to existing large-scale systems unless merited by significant metabolic improvement in the endocrine spheroids.

## Gas-Permeable Culture Platforms: Preventing Anoxia in Endocrine Spheroid Static Culture

A simple and cost-effective solution to providing adequate oxygen in the culture of endocrine and other 3D spheroids while eliminating any effects of shear forces is the use of polydimethylsiloxane culture systems (PDMS). Utilized previously in cell culture, primarily as a hydrophobic and non-adherent substrate, PDMS has recently gained favor due to its improved permeability to gasses, including oxygen and carbon dioxide relevant to cell culture (Harris, 1984; Rosdy et al., 1991; Singhvi et al., 1994; Wang and Deen, 2003). Our group and others have implemented gas permeable static culture systems using PDMS as the basal culture surface for the culture of primary islets of Langerhans and endocrine spheroids derived from stem cells (Papas et al., 2005; Fraker et al., 2007, 2013; Avgoustiniatos et al., 2008; Cechin et al., 2014; Kitzmann et al., 2014). PDMS has reported oxygen permeabilities 100–500 times that of polystyrene (Papas et al., 2005; Zhang, 2006). It is also an inert and biocompatible material that is FDA approved for numerous *in vivo* applications and devices making it ideal for *in vitro* cell culture use.

Early PDMS culture work related to islets was done by the group of Papas, Avgoustiniatos, and Colton. They utilized FEM to interrogate oxygen limitations in conventional islet culture methods for islets of Langerhans (Papas et al., 2005). Their results, confirmed by OCR as a measure of islet viability post-culture, demonstrated that culture on silicone rubber devices completely abrogated islet anoxia at culture densities up to ∼4,400 IEQ/cm^2^, nearly 25 times the standard culture density. One hundred cm^2^ prototypes from Wilson Wolf Manufacturing were implemented with a membrane thickness of 275 μm filled with 500 mL of culture medium. It should be noted that these studies were performed at an incubator pO_2_ corresponding to a fully humidified 95% RA/5% CO_2_ system (142 mmHg) and that the models assumed an IEQ diameter of 150 μm and did not examine the size range distribution of IEQ. This early prototype was the basis for the G-Rex^TM^ technology now broadly implemented in therapeutic immune and stem cell expansion (Bajgain et al., 2014).

One added benefit of the gas permeable basal surface is that the majority of the oxygen is delivered in close proximity to the cells and this eliminates reliance on oxygen diffusion from the apical liquid/air interface. This dependence on diffusion in plastic culture systems limits the amount of culture medium and therefore, the nutrient supply, that can be added without limiting oxygen supply. In PDMS systems, nutrient supply is not limited by this height restriction. While the devices have a higher per unit cost, culture using the PDMS systems is more efficient and cost-effective by reducing the number of needed flasks, the amount of culture medium and labor involved with maintaining cell cultures.

**FIGURE 7:**
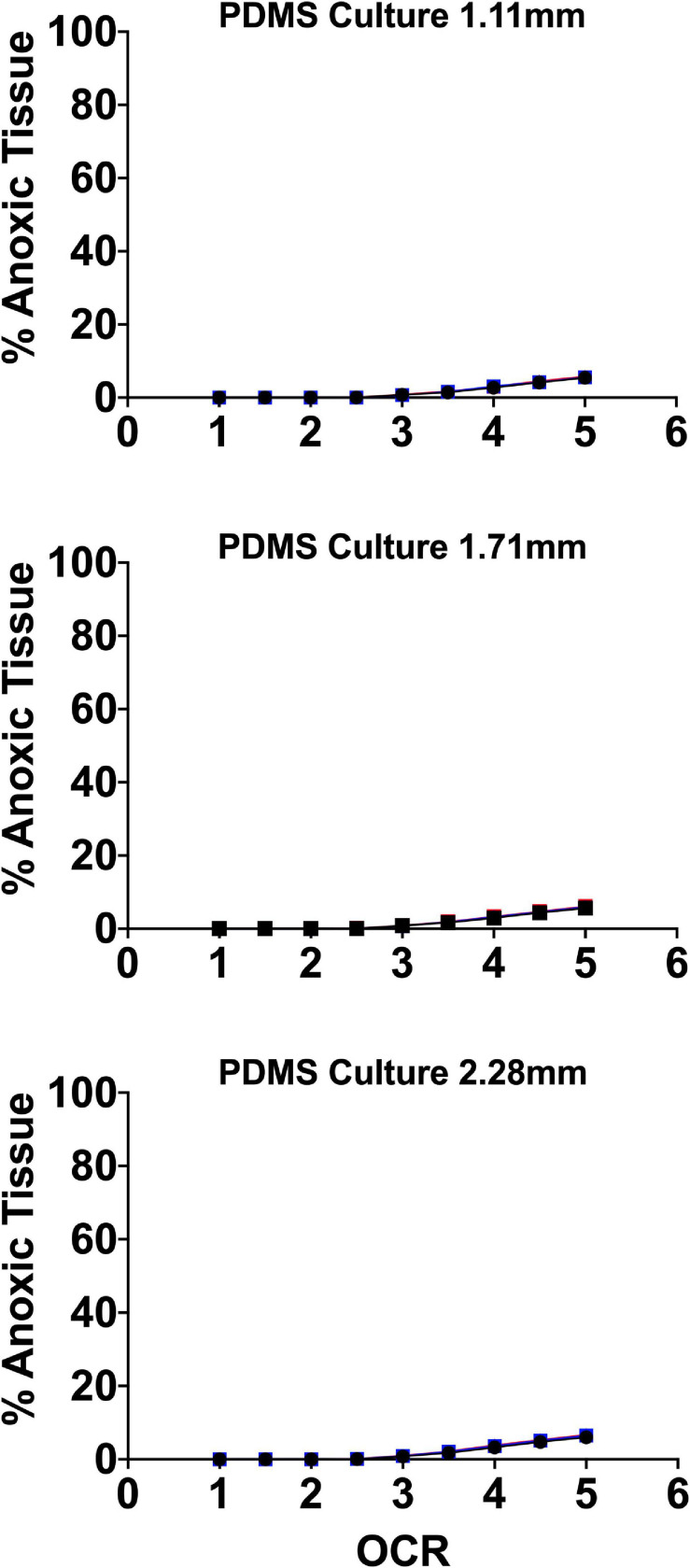
Anoxic tissue volume percentages of endocrine spheroids (representative size range and OCR) cultured on perfluorosilane/PDMS constructs. Anoxia is eliminated in all but the largest aggregate size at the highest oxygen consumption rates. FEM was performed using three seeding densities (2.76–5.05%) and three culture medium heights (1.11, 1.71, and 2.28 mm).

Our group developed a perfluorosilane-impregnated PDMS 275 μm membrane with a honeycomb support structure designed to maximize surface area for gas transport with support sufficient to prevent membrane damage. Given the high affinity of perfluoro-compounds for oxygen, the inclusion of perfluorosilane further improved oxygen mass transfer. Figure 7 shows FEM of the anoxic tissue volume percentage of endocrine clusters of representative size range and OCR cultured on the perfluorosilane/PDMS constructs. FEM was performed, as above, using three different seeding densities and three relevant culture medium heights.

Of note, culture on these platforms all but eliminates anoxia in all spheroid geometries except at elevated OCR. In agreement with the earlier findings of Avgoustiniatos et al. (2008), there is virtually no effect from either seeding density or culture medium height at these settings typically utilized in culture on plastic, where both variables have a significant effect.

Recent advances using PDMS surfaces in the culture of endocrine spheroids include the development of printed microwells for controlled seeding of aggregates in a 96 and 384 well format. These devices have the added benefit of providing oxygen from an increased surface area surrounding the 3D clusters in the wells. These concave wells have been successfully used in the formation of pancreatic clusters from purified beta cells (Lee S.H. et al., 2018).

### Gas-Permeable Platforms Improve Culture and Terminal Differentiation of Murine Pancreatic Buds

Studies performed in the laboratory of Clark Colton using culture of mouse embryonic stem cells on plastic examined the effect of external oxygen concentrations from 0 to 285 mmHg on proliferative capacity and differentiation either +/− the differentiation inhibiting factor, LIF. They observed that oxygen had minimal effect on undifferentiated cell growth and phenotype (+LIF) and posited that it was likely to have a more pronounced effect on cells undergoing differentiation, as we observed (Powers et al., 2008).

In another paper by the same group, they studied the effect of low oxygen culture on murine ES cells, again demonstrating the reduction of spontaneous differentiation but also the loss of pluripotent gene expression. Importantly, they noted that the oxygen level in the gas phase is often quite different from the oxygen level in the microenvironment of the cells and this is neglected in the vast majority of the literature, until recently, making interpretation of results difficult. This can be seen in the FEM models presented above for 2D and 3D culture. They emphasize the importance of tools like FEM to better understand culture conditions (Millman et al., 2009).

Our group examined the role of oxygen in pancreatic development using pancreatic buds from mouse embryoid bodies (Fraker et al., 2007). In our comparison of pancreatic buds cultured on plastic, both at standard (20%) and elevated concentration (35%) to buds cultured on the perfluoro/PDMS composites, the data supported our models. There was both improved proliferation and differentiation in the buds cultured on the perfluoro/PDMS, as well as absence of tissue hypoxia, in contrast with our immunohistochemical observations in samples cultured at either 20% and 35% incubator oxygen concentration. Gene and protein expression of important pancreatic/endocrine/β-cell developmental markers were significantly elevated in the buds cultured on the perfluoro/PDMS constructs as opposed to both normoxic (20%) and hyperoxic (35%) culture on plastic. Most notably, when compared to the *in vivo* endpoint of terminal differentiation in the pancreatic buds, e16.5, gene and protein expression of key pancreatic markers was non-significantly different between the perfluoro/PDMS culture and the *in vivo* counterparts, further confirming the importance of oxygenation in the final stages of pancreatic development, as hypothesized. Also, in examining the expression of pancreatic exocrine markers in this setting, oxygenation promoted endocrine over exocrine differentiation. It should be noted that the size of the buds cultured in higher oxygen levels reached 1 mm and there was no evidence of tissue hypoxia by immune staining indicating that their oxygen consumption rate was likely lower than that of conventional islets of Langerhans.

### Too Much or Too Little: The Goldilocks Paradox of Oxygen Supply

The supply of oxygen to cultured cells/tissues is a delicate balance between anoxia/hypoxia (too little) and oxidant/radical formation from culture medium substrates (too much). While conventional culture occurs at pO_2_ of ∼142 mmHg, vascularized cells in the human body do not typically experience a pO_2_ greater than 100 mmHg and are supplied with sufficient oxygen to avoid sustained hypoxia due to short vascular diffusion distances. With 3D islets/endocrine spheroids, it becomes immediately clear from the above FEM why culture of these aggregates is difficult. Their high metabolic rate and large geometries make it difficult to maintain adequate oxygen supply and impossible to do so without non-physiological pO_2_ gradients across the path of oxygen transfer (surface to core). *In vivo*, islets are thought to experience a nearly uniform tissue pO_2_ of approximately 20–40 mmHg. Therefore, even culture at standard incubator pO_2_ results in apical endocrine spheroid surface exposure to supra-physiological levels and basal surface/core anoxia except in the smallest endocrine spheroid dimensions (50–100 μm). In gas permeable culture platforms in standard incubator settings (142 mmHg), the tissue exposure to supra-physiological levels is even greater.

**FIGURE 8:**
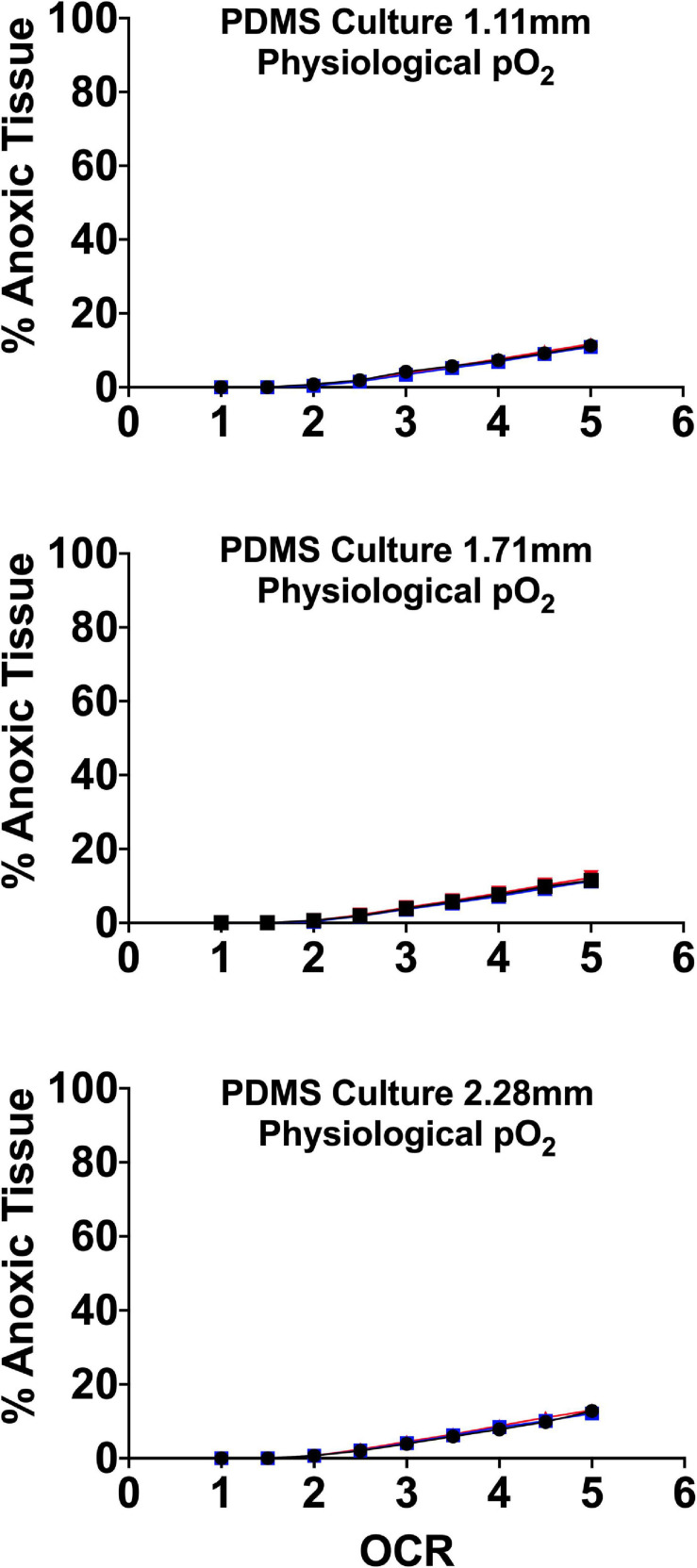
Anoxic tissue volume percentages of endocrine spheroids (representative size range and OCR) cultured on perfluorosilane/PDMS constructs in the presence of physiological pO_2_ (95 mmHg). Anoxia is minimal in all but the largest aggregate size at the highest oxygen consumption rates. FEM was performed using three seeding densities (2.76–5.05%) and three culture medium heights (1.11, 1.71, and 2.28 mm).

There is evidence in the literature that islets are uniquely susceptible to free-radical/oxidant damage due to glucose sensing suppression of superoxide dismutase and therefore prolonged exposure to elevated pO_2_ could be sub-optimal (Gille and Joenje, 1992; Kazzaz et al., 1999; Martens et al., 2005; Pi et al., 2007; Maddalena et al., 2017). For all these reasons, optimizing endocrine spheroid culture requires devices that allow for the maintenance of tissue specific oxygen profiles where core pO_2_ is (1) above the anoxic and functional cutoff noted by Avgoustiniatos and others and (2) below arterial concentrations of ∼100 mmHg (Papas et al., 2005; Buchwald, 2009, 2011). To that end, our group and others have examined the effect of physiologically relevant culture pO_2_ on endocrine spheroid function and viability utilizing gas permeable platforms demonstrating that culturing at more physiologically relevant pO_2_ improves *in vitro* and *in vivo* islet function and viability (Fraker et al., 2013). Culture incubator pO_2_ was set guided by FEM to target a maximum tissue volume maintained within a physiological range of 5–95 mmHg, minimizing “hyperoxia” and anoxia. Figure 8 details the FEM and the subsequent anoxic tissue volume percentage. The models used three seeding densities and culture medium heights, as in prior models, above.

### Gas-Permeable Platforms Improve Human Embryonic Stem (hES) Cell Specification Into Endocrine Fates

As shown in an increasing number of studies, oxygen in culture is recognized as a factor driving both stem cell proliferation and terminal differentiation into endocrine clusters. In 3D aggregates where oxygen gradients are prevalent, lower pO_2_ is associated with proliferative capacity and higher pO_2_ drives terminal differentiation through Notch and HIF-1. As shown from the FEM models, above, it is not feasible to successfully modulate oxygen supply to 3D tissues in plastic culture systems, so gas permeable platforms present a useful tool for the tight control of pO_2_ experienced by culture spheroids.

A paper by Powers et al. (2010) from 2010 described the historical disconnect between ambient incubator pO_2_, pO_2gas_, and the pO_2_ experienced by cells in culture, pO_2cell_, using FEM and mouse embryonic stem cell differentiation into cardiomyocytes on plastic and PDMS culture surfaces to demonstrate how critical cellular oxygen supply is to desired outcome. They used monolayer cultures on polystyrene and functionalized PDMS surfaces implementing FEM to target specific ranges of pO_2cell_ to determine the effect of standard (142 mmHg), physiological (37 mmHg), and hypoxic (7 mmHg) culture on function and viability. Only on PDMS membranes, where pO_2cell_ is equivalent to pO_2gas_ due to the proximity of the gas supply at the basal surface, were functional cardiomyocytes observed. As reported in other cell culture studies on PDMS, including our own, they frequently observed spontaneous aggregation of single cells into larger spheroids. They noted that this resulted in the formation of pO_2_ gradients that had minimal effect on the tissue anoxia of the PDMS group but would have resulted in ∼80% anoxia in spheroids cultured on polystyrene at the highest pO_2_ of 142 mmHg. Unfortunately, the only functional assessment was the observation of spontaneous contraction of the cells, which was observed only on PDMS and polystyrene with a PDMS membrane. The largest number of spontaneously contracting cells were observed on the PDMS surface at a pO_2gas_ of 36 mmHg. This demonstrates the importance of physio-normal pO_2_ (36 vs. 142 mmHg) in terminal differentiation.

During our work with an early group in the SC-β field, BetaLogics, we postulated that the use of gas permeable culture systems to tailor core tissue pO_2_ to that of the physiological niche of pancreatic endocrine tissue (∼20–40 mmHg core tissue pO_2_; 80–100 mmHg incubator pO_2_) would significantly improve terminal differentiation of hES spheroid progenitor clusters into endocrine fates (Cechin et al., 2014). As these clusters were typically in the size range of IEQ (50–400 μm), the culture on PDMS was designed to maintain maximal tissue volume percentage within a physiological range (∼2.7–100 mmHg). Introducing the PDMS culture systems at the last stage of standard differentiation protocols, after they were formed into spheroids, resulted in significantly improved differentiation into insulin and other endocrine hormone positive cells. Analysis by qRT-PCR showed significantly elevated pancreatic gene expression in hES clusters cultured in gas permeable systems relative to controls. *In vitro* production of insulin in response to glucose, improved engraftment and reversal of diabetes *in vivo*, were also observed. Notably, culture in these systems at physiological pO_2_ resulted in dramatically improved segregation of α and β cells, eliminating the challenge of single cell bi-hormonal expression that had plagued other protocols up to that point. This data again confirmed the role of oxygen in endocrine development and function. Given the low oxygen levels (relative to standard oxygen pO_2_) present in the *in vivo* niches of most cell types, it is not surprising that mechanisms such as proliferation, terminal differentiation, and pleiotropic function are increasingly being tied to bioenergetic shifts dependent on oxygen.

## Conclusion and Future Directions

As 3D culture increasingly becomes a preferred mode of physio-mimetic culture, historical culture methods on plastics need to be re-evaluated. Particularly related to tissue oxygenation, the steep gradients that develop from apical to basal surface, both in the culture milieu and in the 3D organoids, lead to hypoxic and anoxic regions, inefficient nutrient metabolism and potentially, shifts in viability, function and gene expression that may deviate in comparison to the same tissue *in vivo.* There is a critical need and a recent surge in device/method research to improve metabolite/nutrient delivery and waste removal in 3D cell cultures. With the emergent data coming from this area of research, it is clear that other variables besides oxygen, such as mechanical forces, growth factors and supportive matrices also play an important role in the culture of organoid systems but metabolic gas exchange has been one of the most challenging obstacles in 3D cell/spheroid research. Given the growing number of therapeutic applications for 3D spheroids, there is a critical need to revisit expansion and differentiation protocols in order to optimize them for their desired end product and application. Much like personalized medicine, individual cell/tissue types have different metabolic and physiological characteristics that are difficult to recapitulate in general culture practices. Like oxygen in the data presented in this work and others, tailoring environmental factors to address the physiological demands of the cultured spheroids can result in a much better *in vitro* model/approximation of the *in vivo* organoid counterpart.

Related to the clinical application of endocrine spheroids, there is much ongoing work to improve/maintain function and viability prior to transplant in an attempt to minimize immune response and accelerate engraftment. Strategies focused on revascularization and co-cultures with supportive mesenchymal stem cells (MSCs) and endothelial precursors (ECs) are making great strides in minimizing post-transplant loss and time to reversal of hyperglycemia. In terms of culture, there is still work to be done to fully understand the role of oxygen in differentiation and function/viability, *in vitro.* Building on past work, minimizing tissue anoxia has a clear effect on transplant immunogenicity and function. With that in mind, combinatorial approaches that slow cellular metabolism (OCR) and maintain tight control of pO_2__*cell*_ could move endocrine spheroid research one-step closer to clinical impact. This could be achieved, as an example, by the use of low temperature culture on gas permeable systems.

Clearly, oxygen is only one of many culture metabolites that plays a role in the viability and function of 2D and 3D cultures. It could be argued that it is the most important given that it has an immediate effect on cell metabolism and the lack of oxygen can rapidly progress to cellular dysfunction and death. A lack of oxygen in cell systems also results in a build-up of toxic waste products that while largely unnoticeable in the larger culture milieu, might have a pronounced detrimental effect in the cell microenvironment. For example, a shift to anaerobic metabolism and glycolysis increases production of acidic byproducts to compensate for loss of the efficiency of energy production using aerobic respiration. This, in turn, can affect both cellular and environmental pH leading to damage beyond the immediate apoptosis/necrosis caused by anoxia. While not the only participant in the viability of cultured cell/tissue biology, oxygen has an undeniably important role in culture approximation of *in vivo* cell/tissue counterparts.

## Author Contributions

CF and GG contributed to the conception of the manuscript. CF, GG, HT, and JD-B reviewed the manuscript and wrote the original draft. GG, HT, and JD-B contributed to the manuscript revision and editing. All authors read and approved the submitted version.

## Conflict of Interest

CF and JD-B have intellectual property/potential financial interests related to the gas permeable culture devices (Oxygen Sandwich) described in the manuscript. The remaining authors declare that the research was conducted in the absence of any commercial or financial relationships that could be construed as a potential conflict of interest.
